# Efficacy of Osthole in Management of Hypoperfused Retina

**DOI:** 10.1155/2018/6178347

**Published:** 2018-03-11

**Authors:** Ran Du, Zhao-yang Meng, Jia-lin Wang, Yan-ling Wang

**Affiliations:** Department of Ophthalmology, Beijing Friendship Hospital, Capital Medical University, Beijing, China

## Abstract

**Purpose:**

To determine the effect of osthole on the retina in a chronic cerebral hypoperfusion (CCH) rat model and to investigate its therapeutic activity.

**Methods:**

Seventy-two rats were randomly allocated into 6 groups. CCH was induced by permanent bilateral common carotid artery occlusion (BCCAO) in five groups. Sham surgery was performed without occlusion of the artery in the sixth group (control group). Animals were administered with saline (model group), osthole (osthole-IG group), aspirin (aspirin group), or ginaton (ginaton group); the osthole-PI group was performed with peribulbar injection of osthole. Four rats in each group were sacrificed every 5 days after drug administration, and histopathology along with morphology of retina were observed. Fundus fluorescein angiography was performed before the animals were sacrificed at day 15. Retinal Akt, NF-*κ*B, Bax, and Bcl-2 levels were assessed using immunohistochemistry, immunofluorescence, and reverse-transcription PCR; retinal injury was assessed using TUNEL in situ; retinal levels of superoxide dismutase (SOD) and malondialdehyde (MDA) were measured.

**Results:**

Fundus fluorescein angiography revealed the retinal vascular diameter in the osthole-IG group rats to be wider than that in the model, osthole-PI, aspirin, or ginaton group rats. Histological analysis of retinal tissue revealed an increase in retinal thickness in all treatment groups, and significant improvement was noticed in the osthole-IG group. TUNEL staining revealed fewer apoptotic cells in the osthole-IG and osthole-PI groups than in the other groups. For immunohistochemistry results, in the osthole-IG group, levels of NF-*κ*B and Akt were lower than those in the other treated groups, while levels of the ratio Bcl-2/Bax were higher. Levels of MDA were lower and levels of SOD were higher in the osthole-IG group than in the other groups.

**Conclusions:**

Osthole protects the retina from ischemia injury secondary to CCH induced by BCCAO, mainly through anti-inflammatory, antioxidant, and antiapoptotic effects.

## 1. Introduction

Chronic cerebral hypoperfusion (CCH) can lead to cognitive impairment and neuronal cell damage, via oxidative stress and inflammation [1]. Carotid artery stenosis (CAS) is currently the most common cause of CCH, and the degree of CAS and its effect on Willis ring directly determine the symptoms of cerebral ischemia. Hypoperfusion of the brain hypoperfusion activates the anaerobic glycolysis pathway in neuron cells which are starved of oxygen. Due to the limited energy production of the anaerobic glycolysis pathway, neurons enter a low-energy pathological state. The ophthalmic artery is the first major branch of the internal carotid artery, and CAS has a direct effect on ocular hemodynamics. This causes slow or even reversed flow in ocular vessels. Ong et al. [2] have reported that in patients with carotid stenosis, the central retinal artery perfusion pressure can be dramatically reduced, to less than 50% of normal values, which in turn can cause retinal ischemia (RI). In CAS, pathological changes are observed in the retinal vessels secondary to RI in up to 29% of patients [3]. Although most are asymptomatic, peripheral retinal hemorrhage and retinal micro aneurysms are not rare. RI can cause blindness, and treatment requires an interdisciplinary approach including ophthalmologists, vascular surgeons, cardiologists, and neurologists, treating ocular complications with laser surgery, intravitreal injections, or antiplatelet therapy, such as aspirin, and involving surgical management including carotid endarterectomy. Less invasive, more effective, and safer treatments are urgently sought. We previously reported that the Rho/MAPK/iNOS pathway was involved in chronic RI, leading to tissue inflammation and apoptosis in a rat model of bilateral common carotid artery occlusion (BCCAO) [4]. Traditional Chinese medicines, for example, ginaton, have also been reported to be beneficial in the management of RI. [5].

The traditional Chinese medicine osthole (7-methoxy-8-(3-methyl-2-butenyl)-coumarin) is a naturally occurring coumarin derivative, which can be derived from *Cnidium monnieri* (L.) Cusson, *Angelica archangelica* L., and imperatorin [6]. Osthole has been traditionally used to treat peripheral neuritis, eczema, and sexual dysfunction, among other conditions [7]. Recently, osthole was reported to possess potent anti-inflammatory [8, 9], antioxidant [10, 11], and antiapoptotic [12–14] properties. The therapeutic effect of osthole in ischemic conditions, such as cerebral ischemia [15, 16], renal ischemia [17], heart transplantation [18], and lung transplantation [19] has also been demonstrated, and administration of 40 mg/kg osthole was reported to ameliorate traumatic brain injury in a rat model.

However, the effect of osthole in RI is still not proven to be beneficial. We hypothesized that the antioxidant and antiapoptotic properties of osthole may be beneficial in RI. In this study, we analyzed the effectiveness of different methods of osthole administration, in comparison to aspirin and ginaton, in a rat model of CCH.

## 2. Materials and Methods

### 2.1. Animals

All protocols were performed in accordance with the Guidance for the Care and Use of Laboratory Animals, issued by the Ministry of Science and Technology of China. All animal procedures were approved by the Institutional Animal Care and Use Committee of Capital Medical University (Beijing, China). The experiments were conducted on 72 adult Brown Norway (BN; 200–250 g) rats, obtained from Capital Medical University. All rats were housed in regular cages in an animal room at 22°C.

### 2.2. Drugs

Osthole (>98% purity) was provided by the National Institute for Food and Drug Control (Beijing, China) and dissolved in a 1 : 9 (*v*/*v*) mixture of Tween 80 and 0.9% sodium chloride. Aspirin and ginaton were provided by the National Institute for Food and Drug Control (Beijing, China) and dissolved in 0.9% sodium chloride.

### 2.3. CCH Rat Model

The CCH rat model was induced using the BCCAO method: All surgical procedures were performed under anesthesia, established by intraperitoneal injection of 1% pentobarbiturate (Sigma Chemical Co., USA) in saline (40 mg/kg body weight). All efforts were made to minimize suffering. A 10 mm median neck incision was made, and the common carotid artery (CCA) was bilaterally separated from the carotid sheath and vagus nerve. Each artery was firmly occluded with 3–0 silk sutures. Incisions were closed with interrupted silk sutures, wounds were cleaned with alcohol, and the animals were allowed to recover for two hours in their cages. Once fully recovered from the anesthesia, the rats were then placed in a humidified container maintained at 36°C.

### 2.4. Experimental Groups

Seventy-two model rats were randomly allocated to 6 groups (*n* = 12 per group): control, model, osthole-IG (intragastric administration), osthole-PI (peribulbar injection), aspirin, and ginaton groups.

Animals assigned to the control group underwent the same surgical procedure without occlusion of the CCA. All other groups underwent the surgical procedure with occlusion of the CCA (model animals).

The osthole-IG group received IG administration of 40 mg/kg of osthole; the aspirin group and the ginaton group also received administration of 40 mg/kg. The osthole-PI group received peribulbar injection of 0.1 ml of 40 mg/kg osthole. The same volume of physiological saline was administered to the control and model groups.

All groups were administered drug or saline twice every day. Electroretinography and fundus photography were performed before the animals were sacrificed. The rat models were humanely sacrificed by injecting an overdose of 1% pentobarbiturate. Four animals in each group were sacrificed on the 5th, 10th and 15th day after the first treatment (*n* = 4/time point/group). One eye was embedded in paraffin, and the other was used for biochemical evaluation.

### 2.5. Fundus Fluorescein Angiography (FFA)

Since preliminary FFA experiments revealed no significant change at both 5 and 10 days, FFA was only performed 15 days after treatment in each group with a scanning laser ophthalmoscope (Heidelberg, Germany) after intravenous injection with Fluorescite (Alcon Laboratories, USA). Vessel diameters were measured in pixels at a 1-disc-diameter distance from the center of the optic disc, as previously described by Hangai et al. [20]. Vessel diameter was calculated as the distance between the half-height points determined separately on each side of the density profile of the vessel image. The average arterial and venous diameters in each rat were recorded by observers masked to each animal's group.

### 2.6. Histopathology and Morphology of Retina

The eyeballs were enucleated, and 7 *μ*m sections were cut along the horizontal/vertical meridian through the optic disc. These sections were fixed in 4% PFA for 1 h at 4°C and then embedded in paraffin followed by cutting 4 *μ*m sections in the vertical meridian through the optic disc. The sections were stained with hematoxylin and eosin and were observed under a light microscope (Leica DM6 B, Germany). Alterations in retinal layer thickness were measured for each eye in the same topographic region of the retina (1 mm from the optic nerve head) under ×400 magnification. For each eye, five sections were measured and then averaged. Cells were counted at ×400 magnification. In each section, three segments were randomly chosen and average counts were presented.

### 2.7. In Situ TUNEL

The terminal deoxynucleotidyl transferase-mediated dUTP (2′-deoxyuridine 5′-triphosphate) nick-end labeling (TUNEL) assay was used to evaluate apoptosis in the retina. The eyes were enucleated, and retinal paraffin sections were prepared as previously described. Apoptosis of the retinal tissue was assessed using the In Situ Cell Death Detection Kit (Merck, Germany) according to the manufacturer's instructions. DAB was used as chromogen. The sections were examined using light microscopy (Leica DM6 B, Germany) under ×400 magnification. Oblique sections were excluded, and the integral optical density (IOD) of TUNEL staining was measured on both sides of the optic nerve head (1 mm from the optic nerve head) using ImagePro 6.2 software (Media Cybernetics Inc., Rockville, USA). The sum of the IOD from 5 sections was calculated, and the mean ± SE of each group was presented.

### 2.8. Double Immunofluorescent Staining

For double immunofluorescent staining of Bax and Bcl-2, the sections were first covered with 3% BSA at 37°C for 30 min and incubated with a rabbit polyclonal antibody against rat Bax (1 : 50, Abcam, USA) at 4°C for overnight in a wet box. After shaking three times in PBS (pH 7.4) on a decolorized shaker for 5 min each, sections were subsequently incubated with Alexa Fluor 594 goat anti-rabbit IgG (1 : 200 dilution, Abcam, USA) at 37°C for 30 min. After rinsing thoroughly in PBS (pH 7.4), the same procedure was repeated with a rabbit polyclonal antibody against rat Bcl-2 (1 : 100, Cell Signaling Technology, USA) and an Alexa Fluor 488 goat anti-rabbit (1 : 200 dilution, Abcam, USA). For dual staining of Akt and NF-*κ*B, the sections were stained with a rabbit polyclonal antibody against rat Akt (1 : 300, Abcam, USA), a rabbit monoclonal antibody against rat NF-*κ*B p50 (1 : 100, Abcam, USA), an Alexa Fluor 594 goat anti-rabbit IgG (1 : 200 dilution, Abcam, USA), and an Alexa Fluor 488 goat anti-rabbit (1 : 200 dilution, Abcam, USA). Sections were visualized using UV excitation wavelength 330–380 nm, emission wavelength 420 nm; FITC green excitation wavelength 465–495 nm, emission wavelength 515–555 nm; and CY3 red light excitation wavelength 510–560 nm, emission wavelength 590 nm with the Nikon Eclipse Ti-SR (Nikon, Tokyo, Japan) and Nikon DS-U3 (Nikon, Tokyo, Japan).

### 2.9. Immunohistochemistry

IHC of paraffin-embedded tissues was performed using the ABC-Staining System according to the manufacturer's instructions (Abcam, USA; Cell Signaling Technology Inc., USA). A rabbit polyclonal antibody against rat Akt (1 : 300, Abcam, USA), a rabbit monoclonal antibody against rat NF-*κ*B p50 (1 : 100, Abcam, USA), a rabbit polyclonal antibody against rat Bax (1 : 50, Abcam, USA), and a rabbit polyclonal antibody against rat Bcl-2 (1 : 100, Cell Signaling Technology, USA) were used as primary antibodies. Primary antibody binding was detected with goat anti-rabbit IgG (1 : 200, Agilent Technologies Inc., USA). Histologic sections were imaged using a microscope (Leica DM6 B, Germany) and a digital camera (Nikon, Japan). IOD measurement was performed as described for TUNEL.

### 2.10. Real-Time Polymerase Chain Reaction (PCR)

Total ribonucleic acid (RNA) of the retinal tissue was extracted using TRIzol reagent (Invitrogen, USA) according to the manufacturer's instructions. RNA (2 *μ*g) was reverse transcribed into first-strand complementary deoxyribonucleic acid (cDNA) (RevertAid First Strand cDNA Synthesis Kit; Thermo Scientific, USA). PCR reactions were performed in the ABI 7500 Detection System using a SYBR green PCR kit (Roche, Switzerland). The primer sets were as follows: NF-*κ*B, 5′-GGTGGAGGCATGTTCGGTAG-3′ (forward), 5′-CGTCATCACTCTTGGCACAATCT-3′ (reverse; accession number NM_008689, http://www.ncbi.nlm.nih.gov/nuccore/NM_008689.2); Akt, 5′-AAGGAGGTCATCGTCGCCAA-3′ (forward), 5′-ACAGCCCGAAGTCCGTTATC-3′ (reverse; accession number NM_009652.3, http://www.ncbi.nlm.nih.gov/nucleotide/260166604); Bax, 5′-GCCTTTTTGCTACAGGGTTTCAT-3′ (forward), 5′-TATTGCTGTCCAGTTCATCTCCA-3′ (reverse; accession number NM_007527, http://www.ncbi.nlm.nih.gov/nuccore/NM_007527.3); Bcl-2, 5′-TGACTTCTCTCGTCGCTACCGT-3′ (forward), 5′-CCTGAAGAGTTCCTCCACCACC-3′ (reverse; accession number NM_009741, http://www.ncbi.nlm.nih.gov/nuccore/NM_009741.4). The parameters were set at 95°C for 1 min and 1 cycle, then 95°C for 15 s, 60°C for 45 s, and 72°C for 45 s and 40 cycles. The fold change in target gene expression was analyzed using the 2-ΔΔCT method.

### 2.11. Measurement of Superoxide Dismutase (SOD) and Malondialdehyde (MDA) Levels in Retina

We determined total SOD activity using the xanthine oxidase method with a commercial assay kit (Nanjing Jiancheng Bioengineering Institute, China), which is based on inhibition of the nitroblue tetrazolium reduction by the xanthine oxidase system as a superoxide generator. We assayed MDA levels in the form of thiobarbituric acid-reacting substances using MDA assay kit (Nanjing Jiancheng Bioengineering Institute, China).

### 2.12. Statistical Analysis

Results are expressed as the mean ± standard deviation. Comparison was performed between groups except the control group which was performed as baseline. Statistical analyses were performed by one-way ANOVA using SPSS 23.0 software (SPSS Inc., Chicago, IL). Homogeneity of variance data were processed by Tukey's test. Heterogeneity of variance data were processed by Dunnett T3 test. Differences were considered statistically significant at *p* < 0.05.

## 3. Results

### 3.1. Objective Examination of Retina

**Figure 1:**
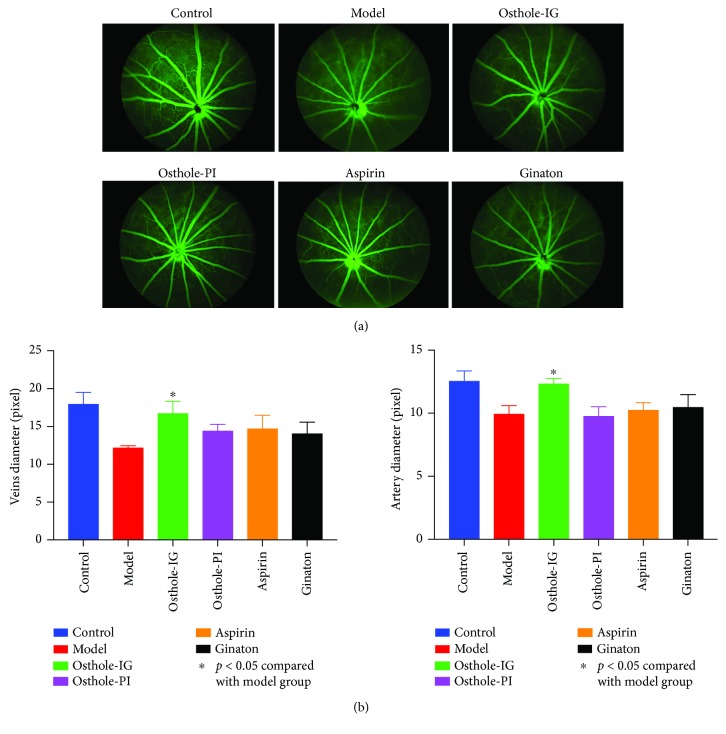
Fundus fluorescein angiography (FFA). FFA images of the retinal vasculature in all six groups. The vasculature of osthole-IG-treated rats was similar to that of control group rats, while significant ischemia was observed in the model group retina. In rats administered with osthole-PI, aspirin and ginaton supply was increased (a). Artery and vein diameters were significantly increased in the osthole-IG group when compared with the model group (*p* < 0.05) and resembled those of the control group (b).

As illustrated in Figure 1(a), FFA revealed that the retinal blood vessel diameter was significantly larger in all four groups that received drugs than in animals administered with saline. CCH-induced reduction in the artery and vein diameter was statistically significant (*p* < 0.05). This decrease was not significantly altered by the administration of osthole-PI, aspirin, and ginaton whereas osthole-IG-induced reversal of vessel diameter was significant which was similar to the values in the control group (Figure 1(b)).

### 3.2. Histological Analysis of Retina

**Figure 2:**
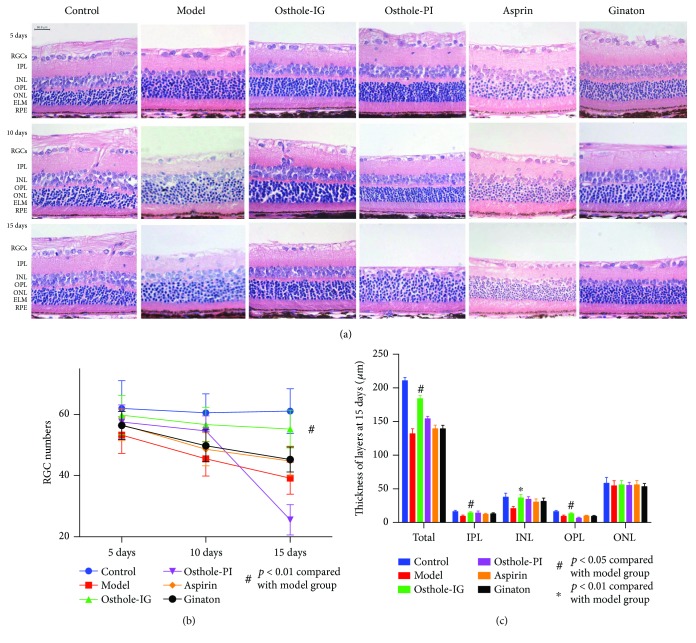
Retina histopathology and morphology results. Retina histopathology and morphology of each group after sacrifice. Scale bar: 50 *μ*m. Retinal thickness in each group at days 5, 10, and 15 after drug administration (a). Bars indicate average thickness ± SEM (*n* = 4). ^∗^*p* < 0.01; ^#^*p* < 0.05. Osthole-IG group retina were thicker than in the other groups at day 15. Osthole-PI group retinal thickness was comparable to the control group retinal thickness at day 5, though the effect was not sustained at day 15. The number of ganglion cells (RGCs) (b) and the thickness of the inner plexiform (IPL), inner nuclear (INL), outer plexiform (OPL), and outer nuclear (ONL) layers also present with the same results (c).

As indicated in Figure 2, surgical CCH induction resulted in thinner retina than what was observed in the control group. Post treatment, the number of retinal ganglion cells (RGCs) in the osthole-PI-treated group was slightly reduced than that in the control group and significantly reduced than that in the other treatment groups at day 15 (*p* > 0.05). In the osthole-IG group, RGCs did not differ significantly from those in the other treated groups at days 5 and 10; however, over the follow-up period of 15 days, this protective effect was significant (*p* < 0.05). Administration of aspirin or ginaton ameliorated retinal ischemic changes and thinning at day 10; however, over the follow-up period of 15 days, this effect was not sustained, unlike the consistent protection provided by osthole-IG. The total thickness of the retina, and the thickness of the inner plexiform (IPL), inner nuclear (INL), outer plexiform (OPL), and outer nuclear (ONL) layers, also exhibited similar trends.

### 3.3. Immunohistochemistry

**Figure 3:**
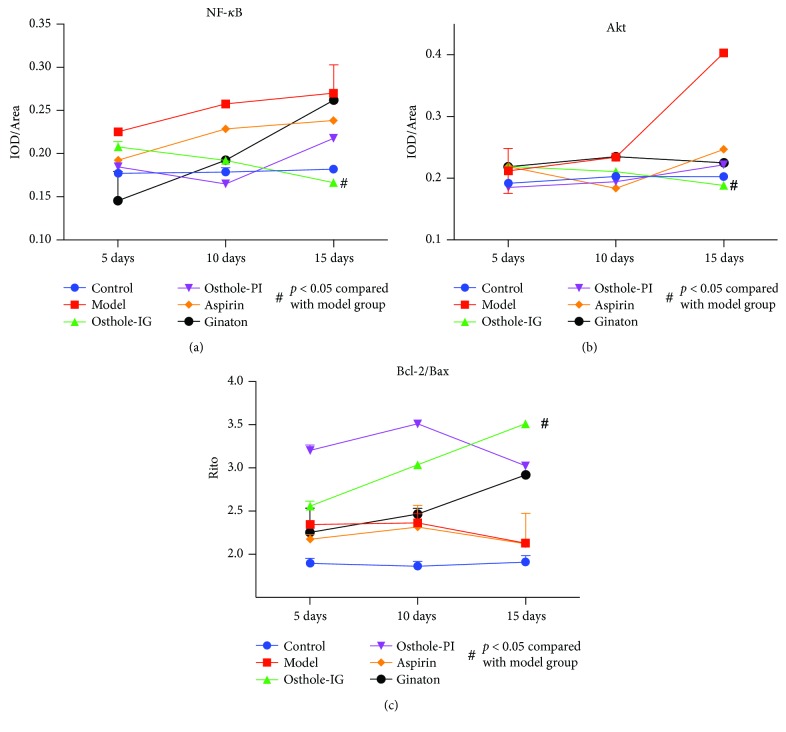
Immunohistochemical staining of NF-*κ*B, Akt, and Bcl-2/Bax. Semiquantitative analysis of NF-*κ*B (a), Akt (b), and Bcl-2/Bax (c) levels by IOD/area. NF-*κ*B expression was significantly lower in the osthole-IG group than in the other groups between days 5 and 15. NF-*κ*B expression was significantly lower in the osthole-PI group than in the model group on day 5 but not on day 15 (a); Akt expressions were significantly lower in the osthole-IG group than in the other groups (b). Bcl-2/Bax expression was significantly higher in the osthole-IG group than in the other groups (c). Parallel changes in Akt staining were observed (b). For the Bcl-2/Bax IOD ratio, the osthole-PI group result was higher than any other group on days 5 and 10 but significantly lower on day 15. In the osthole-IG group, this ratio continued to increase over time. In the other groups, fluctuating results were observed, but the Bcl-2/Bax IOD ratio was lower than that in the osthole-IG group in day 15 (c).

To better understand the effect of these drugs in the retina, we examined the distribution of proteins that were elevated. Histological staining indicated accumulation of NF-*κ*B, Akt, Bcl-2, and Bax in the model group retina, which was not observed in the control retina, but the levels of these proteins appeared to differ between time points in each group (*p* < 0.05). Immunohistochemical slides were analyzed semiquantitatively using integral optical density (IOD) analysis. NF-*κ*B, Akt, Bcl-2, and Bax staining were higher in the CCH than in the control groups (all *p* > 0.05; Figure 3). Levels of NF-*κ*B and Akt in the osthole-IG group were similar to those in the control group and lower than those in the other treatment groups (Figures 3(a)-3(b)); however, only the Akt levels were reduced significantly when compared to the other groups (Figures 3(a)-3(b)), while Bcl-2/Bax levels were highest in this group at day 15 (Figure 3(c)). Aspirin- and ginaton-administered groups exhibited a progressive increase in NF-*κ*B and Akt levels from day 5 to day 15 (Figures 3(a)-3(b)), while these levels did not differ significantly from those in the model group.

### 3.4. Protein Immunofluorescence

**Figure 4:**
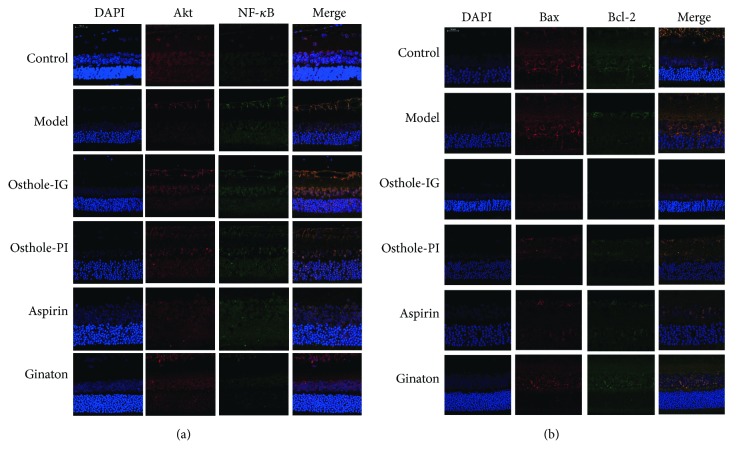
Double immunofluorescent staining of NF-*κ*B, Akt, Bax, and Bcl-2 on the 5th day. NF-*κ*B, Akt, Bax, and Bcl-2 levels on day 5 with double immunofluorescent staining. Akt and NF-*κ*B staining were significantly more prevalent in the model group than in the control group. In addition, NF-*κ*B expression in the osthole-IG group is higher than that in the other treated groups (a). Bax signaling was more prevalent, while Bcl-2 staining was less prevalent, in the osthole-IG group than in the model group. Also, Bcl-2 staining was less prevalent in the osthole-PI, ginaton, and aspirin groups than in the model group but higher than in the osthole-IG group (b).

**Figure 5:**
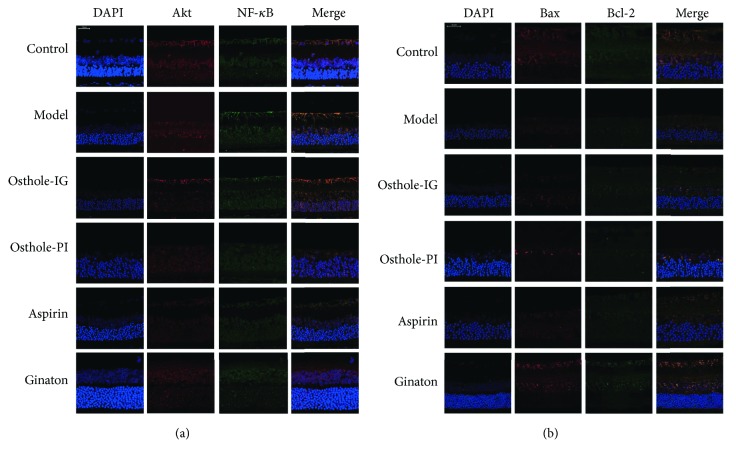
Double immunofluorescent staining of NF-*κ*B, Akt, Bax, and Bcl-2 on the 10th day. NF-*κ*B, Akt, Bax, and Bcl-2 levels on day 10 with double immunofluorescent staining. Levels of Akt and NF-*κ*B remained quite low; Akt and NF-*κ*B staining were significantly higher in the model group than in the control group. In the osthole-IG group, expressions of Akt and NF-*κ*B were similar to those in the control group (a). Bcl-2 was more prevalent in the osthole-IG and ginaton groups, while Bax staining was less prevalent in the osthole-IG and aspirin groups (b).

**Figure 6:**
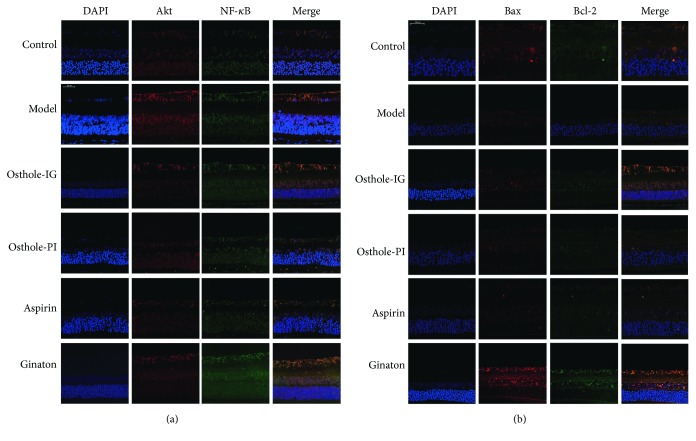
Double immunofluorescent staining of NF-*κ*B, Akt, Bax, and Bcl-2 on the 15th day. NF-*κ*B, Akt, Bax, and Bcl-2 levels on day 15 with double immunofluorescent staining. In the osthole-IG group, expressions of Akt and NF-*κ*B were closer to those in the control group (a). Bcl-2 signaling was more prevalent in the osthole-IG and ginaton groups; Bax staining was less prevalent in the osthole-IG group but higher in the ginaton group (b).

Immunofluorescent staining was used to measure the distribution of proteins in rat retinas. In the control group, levels of Akt and NF-*κ*B remained quite low throughout the study, while Akt and NF-*κ*B staining were significantly more prevalent in the model group. However, staining of NF-*κ*B varied significantly over time, being higher in the osthole-IG group than in the osthole-PI group at 10 days but lower at 15 days (Figures 4–6). Bcl-2 signaling was more prevalent, and Bax staining was less prevalent in the osthole-IG group than in the model group (Figure 4). In addition, Akt and NF-*κ*B staining were less prevalent in the osthole-PI, ginaton, and aspirin groups than in the model group but higher than in the osthole-IG group. The same trend was observed in Bcl-2 staining, and the reverse trend was observed in Bax staining (Figure 5).

### 3.5. In Situ TUNEL

**Figure 7:**
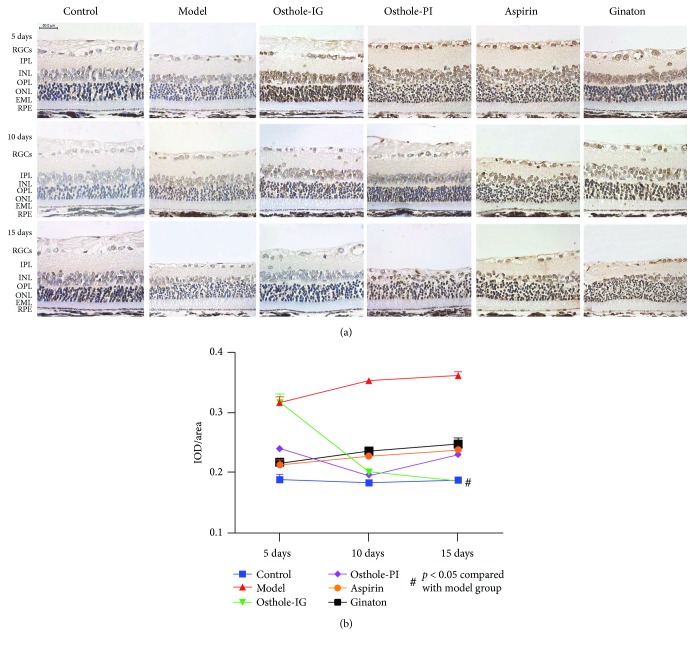
Apoptosis, assessed by TUNEL assay. To evaluate retinal cell apoptosis, TUNEL was preformed (a). Semiquantitative analysis was performed by IOD/area (b). The level of retinal apoptosis was similar in the osthole-IG and model groups at day 5 and higher than in the other treated groups. But from day 10 onwards, less apoptosis was observed in the osthole-IG, and levels were almost decreased to those observed in the control group. In the osthole-PI group, apoptosis increased from day 10.

To evaluate apoptosis, TUNEL (Figure 7) was performed. Apoptosis was more prevalent in the INL and ONL of the retina and in RGCs in all CCH-induced groups as compared to the control group (Figure 7). In the osthole-IG-treated group, apoptosis was significantly reduced than that in the other treatment groups and reaching levels similar to the control group at day 15. The results were analyzed semiquantitatively using integral optical density (IOD) analysis (Figure 7(a)).

### 3.6. Protein Expression in the Retina

**Figure 8:**
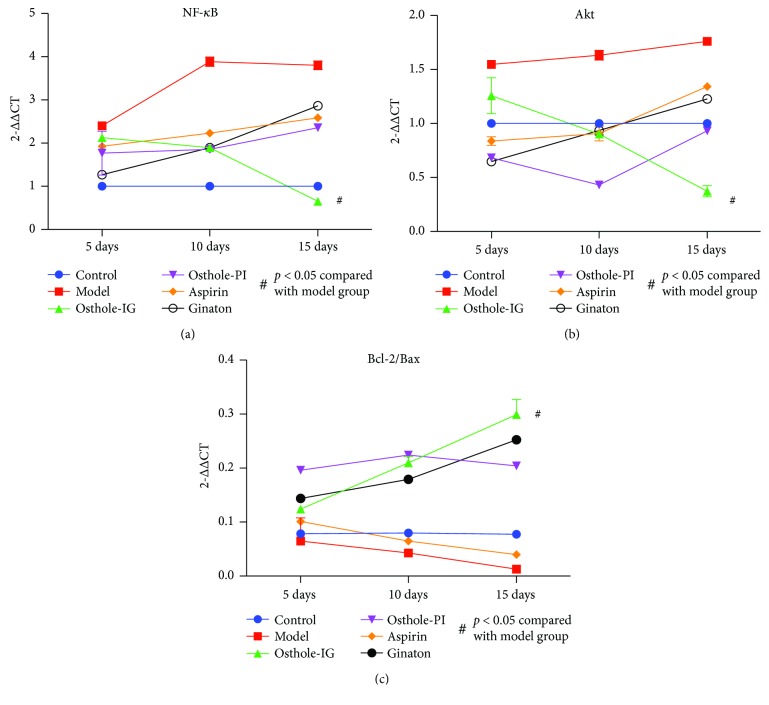
RT-PCR. NF-*κ*B (a), Akt (b), and Bcl-2/Bax (c) mRNA levels in each group at each time point were detected by RT-PCR. Much lower levels of NF-*κ*B and Akt mRNA were observed in the osthole-IG group than in the other treated groups (a, b), while levels of Bcl-2/Bax mRNA were much higher in the osthole-IG group over time (c).

As shown in Figure 7, RT-PCR revealed the Akt and NF-*κ*B levels to be significantly lower in the osthole-IG group than in the other groups and the Bcl-2/Bax ratio to be significantly lower in the osthole-IG group at day 15 than in the control and model groups. As shown in Figure 8, ginaton and aspirin also reduced Akt and NF-*κ*B levels at days 5 and 10, but this efficacy was not sustained at day 15.

### 3.7. MDA Activity and SOD Level

**Figure 9:**
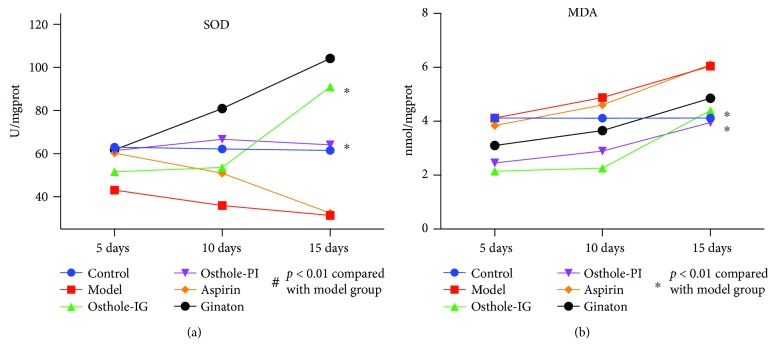
Activities of MDA and SOD in each group. SOD activity was significantly higher in the osthole-IG group than in any other group (a), while MDA activity was lower in the osthole-treated group than in other treated groups and in the model group (b). MDA levels in the osthole-treated groups were closer to those observed in the control group level (b).

To determine the effect of these drugs on lipid peroxidation, we measured retinal levels of MDA. As shown in Figure 9, MDA levels were higher in the CCH retina (*p* < 0.05; Figure 9) than in the control group retinas. Lower MDA activity was detected in the osthole-treated (IG and PI) and ginaton-treated retinas at day 5, which was increased on days 10 and 15, reaching similar levels to the control groups. In the aspirin-treated group, retinal MDA levels were similar to the control group at day 5 but increased to model group levels on day 15.

We also measured SOD and noted that retinal SOD levels were lower in the CCH model (*p* < 0.05) than in the control group. Administration of osthole-IG and ginaton increased SOD levels above control group levels on days 5, 10, and 15, and in rats administered with osthole-PI, SOD levels were equivalent to those in the control group throughout. In aspirin-treated animals, SOD levels declined from days 5 to 10, reaching those of the model group animals on day 15, while the opposite trend was observed in the ginaton group (Figure 9).

## 4. Discussion

At present, over half of cerebral ischemic strokes are caused by CAS [21]. Antiplatelet drugs, such as aspirin, can reduce the risk of stroke and transient ischemic attack [22]; however, alternative therapies are being evaluated for management and reversal of the condition. In this study, we evaluate the protective effect of traditional Chinese medicines, namely, osthole and ginaton in the management of RI. Ginaton is the main component of *Ginkgo biloba* extract, mainly used as a vasodilator, and has been successfully used to treat ischemic conditions of the brain, renal, lung, and heart. Particularly, it has been widely used in treating retinal vein occlusion. Ginaton has been reported to reduce RI secondary to CCH caused by CAS [23]. Osthole, a naturally occurring coumarin derivative, has been reported in dealing with ischemia-reperfusion disease though it has been widely used to treat skin diseases in China [7]. In this study, for the first time, we directly compared administration of PI and GI osthole and ginaton with aspirin in a rat model of RI secondary to CCH.

The BCCAO rat model is widely used in neural impairment, particularly in the brain. Lavinsky et al. reported that in a BCCAO rat model, RGC numbers begin to decline one month after occlusion of the bilateral carotid artery [24]. We used the same rat models, in which CCH was induced, and evaluated the rat eyes within 15 days. Previous studies have reported that 40 mg/kg of osthole has a neuroprotective effect, so we administered this dose in our model [15]. We found that IG administration of osthole improved vessel diameters in the eyes of model rats more effectively than in the osthole-PI, aspirin, or ginaton group (Figure 1). These results indicate that the structure and vasculature of the retina responded better to osthole administered via the IG route. Though osthole-PI, aspirin, and ginaton also improved the retina, improvements did not differ significantly from those observed in the osthole-IG group. Similar results were observed in histological examination, and osthole-IG induced more sustained improvements than other therapies (Figure 2). As reported by Wu et al., osthole may affect neuronal or neuroendocrine function through L-type calcium currents in NG108-15 neuronal cells [25]. This may explain the observed change in RGC numbers after osthole administration.

Osthole has been previously reported to possess anti-inflammatory, antioxidant, and antiapoptotic properties [7]. Chiou et al. in 1933 reported that osthole may block ocular inflammatory effects, particularly in uveitis induced by lens protein, IL-1, and endoxin. However, they did not observe fundus changes after administration of osthole, and the involved pathways remain to be established *in vivo* [26]. The PI3K/Akt pathway regulates cell activities such as apoptosis and inflammatory responses [27]. NF-*κ*B, an important nuclear transcription cell factor in apoptosis and inflammation, is activated by Akt phosphorylation [28, 29]. In order to investigate the mechanism by which osthole improved retina ischemia, we measured Akt and its downstream factor NF-*κ*B (p50), which is a key regulator of cell cycle especially in promoting Bax expression through latent membrane protein 1(LMP-1) [30–33], as indicators of inflammation and apoptosis in model animal retinas (Figure 3–7). Also, we measured the levels of the apoptosis regulators altered by the Akt/NF-*κ*B pathway, Bax, and Bcl-2 (Figure 3–8). Over time, NF-*κ*B and Akt were significantly downregulated in all treatment groups, compared with the untreated model group. Our results are consistent with previous reports of the therapeutic effect of osthole [7].

Furthermore, our results indicate that osthole typically ameliorated oxygen stress as indicated by levels of SOD and MDA which are markers of oxidative stress and an index for lipid peroxidation, respectively. Bilateral artery occlusion can cause increase in free radical concentration in the retina, and we observed a decrease in SOD and increase in MDA levels in the model group suggesting increase in free radicals. However, expression of SOD was increased and MDA was decreased following osthole administration, especially in the osthole-IG group (Figure 9). This result indicates that osthole has a more effective antioxidant function than ginaton and aspirin. Also, we found that osthole irrigation is a more stable and effective modulator of local oxygen stress than peribulbar injection.

Ocular morbidities are most often treated with topical, PI, or systemic medication [5, 33]. In this study, we compared PI and IG administration of osthole and found that while both routes of administration showed improvement in perfusion 5 days after administration, IG administration was more effective than PI administration at reversing histological changes of RI over 15 days. This phenomenon may be explained by the dual blood supply of retina. The ophthalmic artery is a branch of internal carotid artery. Once the CCA is blocked, transport of the drug to the retina is reduced, and hence, the delivery of systemic medications may be reduced. In contrast, PI drug delivery can deliver high concentrations of drug to the retina; however, these concentrations are not sustained, reducing the efficacy of locally delivered drug over time. We postulate that the retinal thinning observed on day 15 in the osthole-PI group may result from direct cytotoxicity of the drug (Figure 2(a)). This observation indicates that PI administration may require lower doses to be administered, in order to avoid potential adverse reactions. Being invasive in nature, PI administration is also typically associated with higher rates of complications than IG administration.

In conclusion, our results indicate that osthole protects against RI injury in a rat model of CCH, exerting anti-inflammatory, antioxidant, and antiapoptotic effects. In addition, we observed that in our rat model study, IG administration of osthole is more effective than PI administration. Furthermore, we found that osthole is more efficient in the management of CCH than ginaton and aspirin. Although our results indicate that osthole may be beneficial in the management of RI, further studies will be required to clarify the optimal vitreous and aqueous concentrations of the drug, and the long-term toxicity of the drug needs to be analyzed. Though the clinical relevance of this animal study is limited, our results provide insight into the pathogenesis of RI secondary to CCH. Further clinical trials will be required to determine how osthole protects against brain ischemia.

## Additional Points

*Animal Experiments*. All applicable ARVO Statements for the care and use of animals were followed. All procedures performed in studies involving animals were approved and monitored by the Institutional Animal Care and Use Committee of Capital Medical University (Beijing, China).
